# Anatomical significance of a posterior horn of medial meniscus: the relationship between its radial tear and cartilage degradation of joint surface

**DOI:** 10.1186/1758-2555-2-1

**Published:** 2010-01-12

**Authors:** Akinori Kan, Midori Oshida, Shigemi Oshida, Masato Imada, Takumi Nakagawa, Shuji Okinaga

**Affiliations:** 1 Department of Sensory & Motor System Medicine, Faculty of Medicine, University of Tokyo, 7-3-1 Hongo, Bunkyo, Tokyo 113-8655, Japan; 2 Department of Orthopedic Surgery, Tokyo Teishin Hospital, 2-14-23 Fujimi, Chiyoda, Tokyo 102-8798, Japan; 3 Department of Legal Medicine, Nihon University School of Medicine, 30-1 Ooyaguchi, Kamimachi Itabashi, Tokyo 173-8610, Japan; 4 Department of Anatomy, Nihon University School of Medicine, 30-1 Ooyaguchi, Kamimachi Itabashi, Tokyo 173-8610, Japan

## Abstract

**Background:**

Traumatic injury and surgical meniscectomy of a medial meniscus are known to cause subsequent knee osteoarthritis. However, the difference in the prevalence of osteoarthritis caused by the individual type of the medial meniscal tear has not been elucidated. The aim of this study was to investigate what type of tear is predominantly responsible for the degradation of articular cartilage in the medial compartment of knee joints.

**Methods:**

Five hundred and forty eight cadaveric knees (290 male and 258 female) were registered in this study. The average age of cadavers at death was 78.8 years old (range: 52-103 years). The knees were macroscopically examined and their medial menisci were classified into four groups according to types of tears: "no tear", "radial tear of posterior horn", "other types of tear" and "worn-out meniscus" groups. The severity of cartilage degradation in their medial compartment of knee joints was evaluated using the international cartilage repair society (ICRS) grading system. We statistically compared the ICRS grades among the groups using Mann-Whitney U test.

**Results:**

The knees were assigned into the four groups: 416 "no tear" knees, 51 "radial tear of posterior horn" knees, 71 "other types of tear" knees, and 10 "worn-out meniscus" knees. The knees with substantial meniscal tears showed the severer ICRS grades of cartilage degradation than those without meniscal tears. In addition, the ICRS grades were significantly severer in the "radial tear of posterior horn" group than in the "other types of tear" group, suggesting that the radial tear of posterior horn in the medial meniscus is one of the risk factors for cartilage degradation of joint surface.

**Conclusions:**

We have clarified the relationship between the radial tear of posterior horn in the medial meniscus and the severer grade of cartilage degradation. This study indicates that the efforts should be made to restore the anatomical role of the posterior horn in keeping the hoop strain, when patients' physical activity levels are high and the tear pattern is simple enough to be securely sutured.

## Background

A meniscus is a crescent-shaped fibrocartilagious structure and has multifunctional roles in normal motion of knee joints [1-3]. It plays roles in proper load transmission, shock absorption, proprioception, and improvements of stability and lubrication of knee joints [4,5]. The meniscus is anchored at the anterior and posterior horns by insertional ligaments which are primarily composed of type I collagen fibrils extending from the main body of the meniscus into the tibial plateau [6]. In particular, the posterior horn, which is stabilized on the tibial plateau in front of the posterior cruciate ligament via insertional ligaments, has a greater thickness than the anterior one, indicating its principal role in a proper localization and stability of the meniscus [7].

A radial tear of posterior horn in the medial meniscus is not rare in the elderly people [8]. However, it is often overlooked by the following reasons. At first, since cartilage degradation is often accompanied with the meniscal tears in the elderly, the knee pain is often explained only by the breakdown of the articular cartilage. Second, incidental meniscal tears on the magnetic resonance (MR) exams of knee joints may mask the substantial radial tear of posterior horn in the medial meniscus [9]. Third, it is technically difficult to visualize the posterior horn of medial meniscus by arthroscopy. The definite diagnosis of the posterior horn's tear of medial meniscus needs precise and detailed arthroscopic examination [10]. If this region was not properly treated, patients' symptoms would persist and some patients would need additional treatments. Thus, the appropriate diagnosis and treatment of the radial tear of posterior horn is an important issue from a clinical perspective.

A meniscus protects the adjacent articular cartilage by keeping the congruency of the femorotibial articulation and distributing axial load properly on the surface of femorotibial articular cartilage [11]. Actually, traumatic meniscal injury or surgical meniscectomy of tear sites in the medial meniscus are well known to cause subsequent progression of knee osteoarthritis [12,13]. However, there has been no report about what type of tear in the medial meniscus is predominantly responsible for the progression of osteoarthritis. The biomechanical significance of meniscal horns is to maintain the hoop strain mechanisms, and the posterior attachment is especially important for this function [14,15]. Hence, we hypothesized that the radial tear of posterior horn would predominantly cause the disruption of the hoop strain and the progression of cartilage degradation. Our specific aim of this study was to investigate the involvement of the radial tear of posterior horn in cartilage degradation of joint surface using a cadaveric study.

## Materials and methods

**Figure 1:**
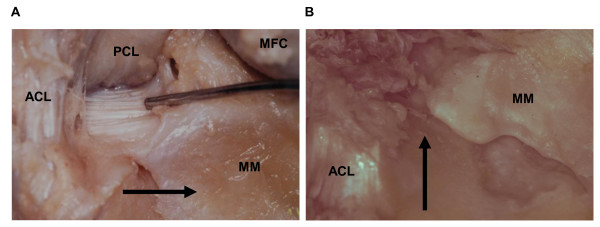
**Posterior horns of medial menisci in cadaveric knees**. A) An intact posterior horn of medial meniscus is shown by a black arrow and an insertional ligament is indicated by a probe. B) A radial tear of posterior horn in the medial meniscus is indicated by a black arrow. ACL, anteritor cruciate ligament; PCL, posterior cruciate ligament; MFC, medial femoral condyle; MM, medial meniscus.

We performed an anatomical study using cadaveric knees. Cadavers who had history of some injuries or surgeries in their knees were excluded from this study. All cadavers were donated to science via the Nihon University after written approval from bereaved families. Five hundred and forty eight knees (290 male and 258 female) were registered in this study. The average age of cadavers at death was 78.8 years old (range: 52-103 years). The meniscal tears were examined macroscopically and were assigned into four groups according to types of tears in the medial meniscus: "no tear" group, "radial tear of posterior horn" group, "other types of tear" group, and "worn-out meniscus" group. "No tear" meant that no substantial tears in the medial menisci were detected by macroscopic examinations. The posterior horn of the medial meniscus is attached to the tibial plateau via the insertional ligament (Figure 1A). The "radial tear of posterior horn" group included knees where macroscopic radial tears were located in the posterior horn's region of the medial meniscus. The representative "radial tear of posterior horn" induced the discontinuity of the medial meniscus at the tear site and the stump of the tear site was able to be observed (Figure 1B). "Other types of tear" group included cases which have no tears in the posterior horn's region of the medial meniscus but have a tear in the other segment of the medial meniscus. Knees with only horizontal or longitudinal tears in the posterior horn are also included in the "other types of tear" group. "Worn-out meniscus" group was defined as the disappeared medial meniscus because of severe degradation. The severity of cartilage degradation in the medial compartment of knee joints was evaluated using the international cartilage repair society (ICRS) grading system [16]. The ICRS grades describe cartilage degradation as follows: grade 0, normal cartilage; grade 1, near-normal cartilage with superficial lesions; grade 2, cartilage with lesions extending to <50% of the depth of the cartilage; grade 3, cartilage with defects that extend to >50% of the depth of the cartilage; and grade 4, severely abnormal cartilage in which the cartilage defects reach subchondral bone. In this study, the cartilage degradation was evaluated in the weight bearing surface of the medial compartment in the knee joints. For statistical analysis, the unpaired *t*-test was used to evaluate the age distribution among groups in the study. Mann-Whitney U test was used to evaluate the relationship between the ICRS grades and types of meniscal tears. In all analyses, a p-value less than 0.05 was considered significant.

## Results

The knees were assigned into four groups according to types of tears in the medial meniscus: 416 "no tear" knees, 51 "radial tear of posterior horn" knees, 71 "other types of tear" knees, and 10 "worn-out meniscus" knees. The average age of cadavers at death in each group was 77.7, 82.3, 82.3 and 80.5 years old, respectively. Although the average age in the "no tear" group was younger than that in the "radial tear of posterior horn" group or the "other types of tear" groups (p < 0.001), there was no significant differences among the "radial tear of posterior horn" group, the "other types of tear" group and the "worn-out meniscus" group. A gender distribution was also comparable among the three groups (Table 1).

**Table 1:** Classification of macroscopic tears of medial menisci in cadaveric knees

	Male	Female	Total (%)	Mean age
**No tear**	249	167	416 (75.9%)	77.7
**Radial tear of posterior horn**	15	36	51 (9.3%)	82.3*
**Other types of tear**	23	48	71 (13.0%)	82.3*
**Worn-out meniscus**	3	7	10 (1.8%)	80.5

**(Total)**	290	258	548	78.8

*: p < 0.001 vs. No tear group

The cartilage degradation of medial compartment was compared using the ICRS grading system among the four groups. The knees with no meniscal tears revealed 288 grade 0 or 1, 62 grade 2, 50 grade 3, and 16 grade 4 ICRS grades of cartilage degradation in their medial compartments. The "radial tear of posterior horn" group was 4, 3, 17 and 27 cases, the "other types of tear" group was 15, 9, 22 and 25 cases, and the "worn-out meniscus" group was 0, 1, 1 and 8 cases, respectively (Table 2). It revealed that the knees with substantial meniscal tears were susceptible to the severer grades of cartilage degradation than those without meniscal tears (p < 0.001). More importantly, the "radial tear of posterior horn" group exhibited the significantly severer ICRS grades than "other types of tear" group (p < 0.05). The "worn-out meniscus" group also showed the severer grades of cartilage degradation than "other types of tear" group (p < 0.05).

**Table 2:** The relationship between macroscopic tears of medial menisci and cartilage degradation

ICRS grade	<1°	2°	3°	4°
**No tear**	288	62	50	16
**Radial tear of posterior horn**	4	3	17	27*#
**Other types of tear**	15	9	22	25*
**Worn-out meniscus**	0	1	1	8*#

*: p < 0.001 vs. No tear group #: p < 0.05 vs. Other types of tear group

## Discussion

We demonstrated that the radial tear of posterior horn in the elderly was associated with the severer grade of cartilage degradation by the cadaveric study. Our data showed that knees with substantial meniscal tears were susceptible to the severer cartilage degradation than those with no tears, although this may partly be explained by the fact that the average age in the "no tear" group was younger than that in the other groups. On the other hand, since the age and gender distributions among the "radial tear of posterior horn", "other types of tear" and "worn-out meniscus" groups were comparable, we can precisely compare the prevalence of cartilage degradation among the three groups. As a result, the "radial tear of posterior horn" group exhibited the severer ICRS grades than "other types of tear" group, indicating a predominant involvement of the radial tear of posterior horn in cartilage degradation.

However, there are several limitations in this study. At first, we have only evaluated degrees of cartilage degradation on the articular surface without taking account of the existence of osteophyte, which is one of the considerable factors of knee osteoarthritis. Therefore, the prevalence of osteoarthritis in this study may be lower than that of radiographic osteoarthritis evaluated by the conventional classification using Kellgren-Laurence grading system [17]. Second, we evaluated the meniscal tear by a macroscopic examination. It was reported that incidental meniscal findings on MR exams of the knee are common in the general population and increase with age [9]. It is possible that the small tear or degradation inside the body of the meniscus could not be detected in this study. Third, knee osteoarthritis might have a certain correlation with the degradation of lateral meniscus as well as that of medial meniscus. Since we focused on the relationship between the types of medial meniscal tears and medial compartmental cartilage degradation, we did not investigate the types of lateral meniscal tears and we cannot exclude the involvement of some specific type of lateral meniscal injury in the cartilage degradation. Finally, although this study showed the significant relationship between the radial tear of posterior horn and high incidence of cartilage degradation, further studies needs to be investigated to clarify their cause-result relationship between the radial tear of posterior horn and cartilage degradation.

Our current study suggests the anatomical importance of the posterior horn of medial meniscus. Hence, we must carefully check the posterior horns during arthroscopic examinations and try to repair the tear site and maintain the function as possible. There are several reports on the arthroscopic repair of the posterior horn's injury in the medial meniscus [18-20]. We are sure that the efforts should be made to restore the important function of posterior horn and maintain the hoop strain, when patients' physical activity levels are high and the tear types are simple enough to be securely sutured.

The current study is the first report to address the involvement of the radial tear of posterior horn in cartilage degradation of joint surface by a human cadaveric study. To further investigate the anatomical significance of the posterior horn of medial meniscus will lead the correct understanding of the knee kinematics and the improvement in treatments of its tear.

## Conclusions

We have clarified that there is a significant relationship between the radial tear of posterior horn and the cartilage degradation of joint surface.

## Competing interests

The authors declare that they have no competing interests.

## Authors' contributions

AK participated in analyzing the data and drafting the manuscript. MO, SO, MI and SO participated in the design of the study and collect the data. TN revised the manuscript critically for important intellectual content. All authors read and approved the final manuscript.
